# The new impact factor of International Brazilian Journal of Urology is 1.342. Where can we get?

**DOI:** 10.1590/S1677-5538.IBJU.2020.06.01

**Published:** 2020-09-02

**Authors:** Luciano A. Favorito

**Affiliations:** 1 Unidade de Pesquisa Urogenital Universidade do Estado de Rio de Janeiro Rio de JaneiroRJ Brasil Unidade de Pesquisa Urogenital - Universidade do Estado de Rio de Janeiro - Uerj, Rio de Janeiro, RJ, Brasil,; 2 Serviço de Urologia Hospital Federal da Lagoa Rio de JaneiroRJ Brasil Serviço de Urologia, Hospital Federal da Lagoa, Rio de Janeiro, RJ, Brasil

## COMMENT

In June 2020 the impact factor of International Brazilian Journal of Urology rises to 1.342. This is the second biggest impact in its history. The journal impact factor is a metric that reflects the yearly average number of citations that articles published in the last two years in a given journal received. The impact factor is very important to the journal importance evaluation. This new impact is due to the hard work of the entire team o Int Braz J Urol and our goal will be to make the Int Braz J Urol impact rise even further and will place the International Brazilian Journal of Urology as one of the five most important in the area at the end of our management.

The November-December number of *Int Braz J Urol* , the 6th under my supervision, presents original contributions with a lot of interesting papers in different fields: Prostate Cancer, Male Infertility, Renal Cell Carcinoma, Urinary Diversion, Hypospadia, Urinary Stones, Ureteral Cancer, Erectile dysfunction, Testicular Torsion, Prostate Biopsy, Partial Nephrectomy, Hypospadias and Covid-19 in Urology. The papers came from many different countries such as Brazil, USA, Serbia Turkey, China, France, Italy, India and Romania, and as usual the editor´s comment highlights some of them.

In the present issue we present three important papers about Renal and Ureteral Stones. Dr. Wang and colleagues from China performed in page 902 ( 1 ) a nice systematic review about the surgical treatments for proximal ureteral stones > 10mm comparing various surgical options such as extracorporeal shock wave lithotripsy (ESWL), ureteroscopic lithotripsy (URSL), percutaneous nephrolithotomy (PCNL) and laparoscopic ureterolithotomy (LU) and concluded that LU have the potential to be considered as the first treatment choice of proximal ureteral stone ≥10mm. Drs. Torricelli and Monga from Brazil and USA ( 2 ) present in page 927 a nice narrative review about the Staghorn renal stones and concluded that this stones are most of times composed of struvite and related to urinary tract infection. Careful preoperative planning is essential to achieve stone-free status. PCNL is the treatment of choice and auxiliary procedures such as SWL and flexible ureteroscopy should be used to treat residual fragments. Both prone and supine are effective. The goals of the treatment are the complete absence of kidney stones and eradication of infection with antibiotics and Close follow-up is advised with regular imaging exams and urine culture and Dr. Sahan and collegues from Turkey presented in page 1010 ( 3 ) a prospective randomized study about the flexible ureterorenoscopy (f-URS) and laser lithotripsy with regional anesthesia vs general anesthesia and concluded that both general anesthesia and regional anesthesia are equally effective and safe anesthesia methods for f-URS procedures. However, regional anesthesia group showed significantly increased likelihood of bradycardia and mucosal injury during surgery, and significantly decreased surgeon comfort during surgery. The editor in chief would like to highlight the following works too:

Dr. Wang and collegues from China ( 4 ) on page 934 evaluated the efficiency of an energy density of 0.05mj/mm2 of low intensity extracorporeal shockwave therapy (Li-ESWT) on erectile dysfunction (ED) patients and concluded that the energy flux density (EFD) of 0.05 of Li-ESWT could improve the erectile function of ED patients with PDE5i response. In addition, EFD of 0.05 of Li-ESWT treatment could turn PDE5i non responders to responders.

Dr. Alger and collegues ( 5 ) from USA performed on page 962 an interesting study about the impact of obesity on perioperative outcomes and urethral stricture recurrence after anterior urethroplasty and concluded that despite the association with increased urethral stricture length and estimated blood loss, obesity is not predictive of adverse perioperative outcomes or stricture recurrence. Obese patients should be offered urethral reconstruction, but patient selection and preoperative counseling remain imperative.

Dr. Dias Filho and collegues ( 6 ) from Brazil performed on page 972 an interesting study about the presentation delay, misdiagnosis rate, inter-hospital transfer times and testicular salvage for testicular torsion patients treated in our state’s public health system and concluded that the low overall testicular salvage rates originated from a large proportion of late presentations combined with long transfer times caused by frequent misdiagnoses. The authors results indicate that efforts to improve salvage rates should aim at enhancing population-wide disease awareness and continuously updating physicians working at primary and secondary levels-of-care about scrotal emergencies.

Dr. Sivaraman and collegues ( 7 ) from Italy developed on page 984 an interesting study about focal therapy (FT) for localized prostate cancer (PCa) treatment is raising interest and concluded that HIFU FT guided by MRI-US fusion may allow improved functional outcomes and fewer complications compared to US-guided HIFU FT alone.

Dr. Sefik and collegues ( 8 ) from Turkey analyzed on page 993 the course of anxiety and depression before and after transrectal ultrasound-guided prostate biopsy (TRUS-Bx) and in the postoperative 1st month when the histopathological biopsy result was obtained and concluded that pre-biopsy anxiety disappeared after BX, but there was a significant increase in anxiety and depression in patients after the diagnosis of malignancy.

Dr. Zidde and collegues from Brazil ( 9 ) performed an interesting translational study on page 1021 (the cover paper in this number) about the arterial segments of ovine kidney and analyze arterial injuries caused by simulated partial nephrectomy of cranial pole. The authors concluded that the segmental distribution of renal artery, the proportional volume of each segment and arterial injuries after cranial pole resection in ovine kidneys are different from what is observed in human kidneys. Meanwhile, ovine kidneys show a primary segmental division on anterior and posterior, as in humans, but different from swine. These anatomical characteristics should be considered when using ovine as animal models for renal experimental and/or training procedures.

Dr. Bandinni and collegues from Serbia, Romania, India and Italy ( 10 ) performed on page 1029 evaluated the feasibility of vacuum physiotherapy meant to decrease graft contraction and recurrent penile curvature (PC), hence successful tubularization and a straight penis in patients underwent two-stage buccal mucosa graft (BMG) urethroplasty, in proximal hypospadias repair and concluded that physiotherapy with the vacuum device is safe, easy and practically feasible. Our vacuum physiotherapy protocol had high compliance rate. Vacuum physiotherapy should be considered for further assessment in patients undergoing two stage hypospadias repair using buccal mucosa.

Dr. Gomes and collegues from Brazil ( 11 ) on page 1042 evaluated the impact of COVID-19 on clinical practice, income, health and lifestyle behavior of Brazilian urologists during the month of April 2020 and concluded that COVID-19 produced massive disturbances in Brazilian urologists’ practice, with major reductions in patient visits and surgical procedures. Distressing consequences were also observed on physicians’ income, health and personal lives. These findings are probably applicable to other medical specialties.

Dr. Macedo and collegues from Brazil ( 12 ) performed on page 1072 an amazing alternative procedure for distal hypospadias consisting of urethral mobilization and partial glandar disassembly, namely GUD (glandar urethral disassembly) technique and that this operation can be regarded as a genuine alter- native to distal hypospadias (coronal and subcoronal) but should not be addressed to midshaft forms.

The Editor-in-chief expects everyone to enjoy reading and for sure better times will come soon.
